# Apolipoprotein E (APOE) and Alzheimer’s disease risk in a Ugandan population: A pilot case-control study

**DOI:** 10.1097/MD.0000000000042407

**Published:** 2025-05-09

**Authors:** Kamada Lwere, Haruna Muwonge, Hakim Sendagire, Martha Sajatovic, Joy Louise Gumukiriza-Onoria, Denis Buwembo, William Buwembo, Rita Nassanga, Rheem Nakimbugwe, Aisha Nazziwa, Ian Guyton Munabi, Noeline Nakasujja, Mark Kaddumukasa

**Affiliations:** a Department of Microbiology, School of Biomedical Sciences, College of Health Sciences, Makerere University, Kampala, Uganda; b Department of Microbiology, Faculty of Health Sciences, Soroti University, Soroti, Uganda; c Habib Medical School, Faculty of Health Sciences, Islamic University in Uganda, Kampala, Uganda; d Department of Physiology, School of Biomedical Sciences, College of Health Sciences, Makerere University, Kampala, Uganda; e Neurological and Behavioral Outcomes Center, University Hospitals Cleveland Medical Center, Case Western Reserve University School of Medicine, Cleveland, OH; f Department of Psychiatry, School of Medicine, College of Health Sciences, Makerere University, Kampala, Uganda; g School of Public Health, College of Health Sciences, Makerere University, Kampala, Uganda; h Department of Anatomy, School of Biomedical Sciences, College of Health Sciences, Makerere University, Kampala, Uganda; i Department of Radiology, School of Medicine, College of Health Sciences, Makerere University, Kampala, Uganda; j Department of Medicine, School of Medicine, College of Health Sciences, Makerere University, Kampala, Uganda.

**Keywords:** Alzheimer’s disease, APOE, case-control study, Uganda

## Abstract

Alzheimer’s disease (AD) is a neurodegenerative disorder that is characterized by cognitive decline and progressive functional impairment. The Apolipoprotein E (APOE) gene, particularly its ε2, ε3, and ε4 alleles, plays a crucial role in lipid metabolism, and has been implicated in AD pathogenesis. Although the APOE ε4 status is associated with an increased risk of AD, its impact varies across populations. This study investigated the prevalence of and association between APOE alleles and AD risk in a Ugandan cohort. This case-control study was conducted in Uganda, and included 87 participants (45 patients with AD and 42 healthy controls). Cognitive assessment was performed using the Montreal Cognitive Assessment (MoCA) and clinical diagnoses were based on the ICD-11 and DSM-5 criteria. Venous blood was collected for APOE genotyping by polymerase chain reaction. Statistical analyses, including logistic regression and generalized additive models (GAMs), were used to assess the association between APOE alleles and AD risk after adjusting for age, education, and sex. This study included 45 patients with AD and 42 healthy controls. The AD group was significantly older than controls (79.6 vs 73.0 years; *P* = .0006). The ε4 allele was common in both the AD (42.2%) and control groups (44.0%), which was higher than the 1000 Genomes African ancestry data. No significant association was found between the APOE genotype or allele dosage and AD risk after adjusting for age, sex, and education. However, the probability of AD increases with age, particularly among ε4 carriers with lower educational levels. While APOE ε4 status was associated with a higher predicted probability of AD in older adults, no statistically significant relationship was observed in the Ugandan cohort. These findings support the need for larger population-specific studies to explore APOE’s role of APOE in AD risk across sub-Saharan Africa.

## 1. Introduction

### 1.1. Background

Alzheimer’s disease (AD), a neurodegenerative disorder affecting over 50 million people worldwide,^[1]^ is characterized by the accumulation of amyloid-beta (Aβ) plaques and neurofibrillary tangles in the brain, leading to cognitive decline, behavioral changes, and progressive functional impairment. The complex etiology of AD is driven by a multifaceted interplay between genetic and environmental factors such as diet, physical activity, exposure to toxins, and cardiovascular health, underscoring the need for a deeper understanding of its genetic underpinnings to develop targeted interventions.^[2]^

Among the genetic factors implicated in AD, the Apolipoprotein E (APOE) is a crucial determinant, particularly because of its role in lipid transport and metabolism in the brain, which is essential for maintaining neuronal function and integrity.^[3–5]^ Disruptions in lipid transport can lead to impaired membrane repair, synaptic dysfunction, and increased amyloid-beta accumulation, all of which contribute to AD pathogenesis.^[6]^ APOE has three common isoforms, ε2, ε3, and ε4, each of which is associated with different levels of AD risk.^[7]^ The ε4 allele is linked to an increased risk of AD and an earlier onset of symptoms, likely because of its role in promoting Aβ aggregation and impairing Aβ clearance through its interaction with receptors such as low-density lipoprotein (LDL) receptor-related protein-1 (LRP1), which are involved in Aβ uptake and degradation, as well as affecting cellular pathways that regulate Aβ metabolism.^[8]^ Conversely, the ε2 allele appears to offer protection against AD, potentially by enhancing Aβ clearance and reducing neuroinflammation.^[9]^ The ε3 allele, which is often considered neutral, may provide a relatively protective effect compared to ε4, meaning that while it does not actively increase AD risk like ε4, it does not offer the same level of protection as ε2.^[10]^

The pathology of AD in relation to APOE is further complicated by isoform-specific interactions with other pathological proteins such as tau. APOE4 not only influences the deposition and aggregation of Aβ but also exacerbates tau pathology and associated neurodegeneration.^[11,12]^ Moreover, APOE4 has been implicated in the dysfunction of several critical brain processes, including synaptic integrity, glucose metabolism, and cerebrovascular function.^[13]^ The multifaceted involvement of APOE in AD pathogenesis makes it a pivotal target for therapeutic strategies aimed at delaying or mitigating AD progression.

Emerging research suggests that the differential effects of APOE isoforms on lipid metabolism and receptor interactions in the brain may also contribute to AD pathology. For example, APOE4 interacts with receptors such as LDL receptor-related protein 1 (LRP1) and very-low-density lipoprotein (VLDL) receptors more strongly than APOE2 or APOE3, leading to altered lipid uptake and impaired clearance of amyloid-beta.^[14]^ Conversely, APOE2 is associated with enhanced receptor-mediated lipid transport and neuroprotection.^[9]^ APOE4’s interaction with LDL receptor-related proteins, such as LRP1 and VLDL, alters lipid transport and distribution, potentially exacerbating AD-related processes. Understanding these isoform-specific mechanisms is crucial for developing targeted interventions to modify the disease course.

Although extensive research has been conducted on the effect of APOE ε4 allele on AD, its effects vary considerably across populations. In African populations, particularly in sub-Saharan Africa, the association between APOE ε4 and AD is less consistent than that in other regions. For example, studies have shown a well-established link between APOE ε4 and AD in Africa, but this association is either absent or markedly weaker in sub-Saharan Africa.^[15]^ These geographical disparities suggest a complex interplay of gene-environment interactions that may influence AD pathogenesis differently in various regions. For example, environmental factors specific to sub-Saharan Africa, such as diet, infectious disease prevalence, and limited access to healthcare, may play a significant role in modulating AD risk in these populations^[16]^

The variability in the impact of APOE ε4 on AD across African populations could be attributed to unique genetic variants or environmental factors that modulate AD risk differently than in other global populations.^[17–19]^ For instance, earlier studies have suggested a significant correlation between APOE ε4 homozygosity and AD in certain populations such as Yoruba.^[20,21]^ This diversity underscores the importance of expanding genetic studies in underrepresented populations to uncover novel insights that could provide more effective and personalized treatments for AD.

Despite the significant contribution of the APOE genotype to AD risk, age of onset, and response to treatment, most research has concentrated on European or Asian populations, leaving a substantial knowledge gap regarding APOE’s influence of APOE on AD in genetically diverse African populations. This study aimed to bridge this gap by investigating the frequency and impact of the APOE alleles (ε2, ε3, and ε4) in older adults with AD in Uganda, a country with a unique genetic background and environmental context. This study may provide valuable insights into the diverse genetic factors that influence AD risk in African populations. By focusing on an Ugandan cohort, this study aimed to provide valuable insights into the genetic epidemiology of AD in sub-Saharan Africa, with potential implications for the development of targeted interventions for this unique population.

## 2. Methods

### 2.1. Study design and setting

This case-control study was conducted in the rural-urban District of Uganda, a region noted for its diverse demographic composition, encompassing urban, suburban, and rural areas, and housing a population of approximately two million.^[22]^ Individuals aged ≥ 60 years were enrolled in the study. Recruitment involved community leaders, such as Local Councilors and Village Health Teams, who used their local knowledge to identify and contact potential participants at their residences. The AD group was selected from a previous dementia study cohort of 500 elderly individuals from Nansana and Busukuma, representing both urban and rural areas.^[23]^

### 2.2. Clinical assessment and diagnoses

Cognitive screening was performed by trained interviewers using the Montreal Cognitive Assessment (MoCA). The MoCA cutoff values were set at ≥25 for normal controls, 18 to 25 for Mild cognitive impairment (MCI), and ≤17 for dementia.^[24,25]^ The clinical evaluation was performed by a psychiatrist who adhered to the ICD-11 and DSM-5 criteria. Participants were classified as normal, mild cognitive impairment (MCI), or AD based on a consensus clinical diagnosis by two psychiatrists and a neuropsychologist.^[26,27]^ The diagnostic process began with an initial assessment of the patient’s symptoms and concerns, gathering medical history, family mental health history, and previous treatments or diagnoses.

Cognitively healthy controls were mostly participants’ spouses, to ensure similarity in diet and environment. The research assistants collected the participants’ demographic data such as birth date, education level, and past occupation, while the medical histories of diabetes, heart disease, and hypertension were gathered via structured questionnaires. The exclusion criteria for both cases and controls were major psychiatric disorders and significant neurological conditions in addition to dementia. Participants with severe systemic diseases such as chronic kidney disease, sepsis, heart failure, diabetes, and recent antibiotic use within six weeks were also excluded.

Educational attainment was assessed during the participant interviews by recording the highest level of formal education. Participants were asked to report their completed education, which was classified into the following categories: no formal education, primary education, secondary education, and tertiary education (including vocational training, college, or university degrees).

For statistical analyses, education was treated as a continuous variable, representing the total number of years of formal schooling. This variable was included as a covariate in the logistic regression models to adjust for the potential confounding effects of education on cognitive status and APOE genotype associations.

### 2.3. Sample size estimation

The sample size was calculated using the G*Power software (version 3.1), assuming a chi-square test for independence. We selected an effect size (w) of 0.35, representing a moderate association. Prior studies have reported a moderately increased risk of AD associated with the APOE ε4 allele in African ancestry populations, including African Americans. For example, a study in 2014 reported a significant but moderate association between ε4 carrier status and incident AD in African Americans, with similar trends observed in other populations of African descent.^[20]^ Given the variability in reported effect sizes and the limited data from sub-Saharan African cohorts, we selected an effect size of 0.35, representing a moderate association, to ensure that our sample size was both statistically sound and feasible for this pilot case-control study. This effect size was chosen to reflect a conservative estimate, while remaining feasible for recruitment within the context of this pilot study. The significance level (α) was set at 0.05 (two-tailed), with a power (1 − β) of 0.80, and an allocation ratio of 1:1 between the cases and controls. The minimum sample size required was estimated to be 80 participants (40 cases and 40 controls), providing sufficient power to detect a moderate association between APOE alleles and AD risk in this population.

### 2.4. Efforts to minimize bias

To reduce potential confounding by environmental factors, controls were primarily selected from the participants’ spouses, ensuring greater similarity in lifestyle, diet, and living conditions. Cognitive assessments were performed using the MoCA with established cutoff scores, while clinical diagnoses adhered to the ICD-11 and DSM-5 criteria. This standardized approach minimizes the risk of diagnostic bias.

Recruitment was conducted in partnership with local council leaders and Village Health Teams, leveraging their knowledge of the community to achieve broad representation across urban, suburban, and rural settings, thereby reducing selection bias. To further address the risk of genetic relatedness within and between the case and control groups, the recruitment team actively screened participants to exclude close biological relatives (e.g., siblings, parent-offspring, or first cousins). This was achieved through structured interviews and a careful review of family histories.

### 2.5. Ethical considerations

Informed consent was obtained from all participants directly or from their proxies when necessary, to ensure voluntary participation and understanding of the study’s objectives and procedures. All procedures undertaken in this study were conducted in accordance with the ethical guidelines set forth by the research and ethics committee as well as the 1964 Helsinki Declaration and its subsequent amendments and comparable ethical standards. The study protocol was reviewed and approved by the School of Biomedical Sciences Research and Ethics Committee of Makerere University (approval number SBS-2022-256) and Uganda National Council of Science and Technology (approval number HS2930ES).

### 2.6. Sample collection and genotyping

Venous blood (7 mL) was collected from all participants and stored at −4°C until analysis. Genotyping of APOE alleles was performed using polymerase chain reaction (PCR), as previously described.^[28]^

### 2.7. Human genomic DNA

Genomic DNA was extracted from human whole blood samples using the QIAamp DNA Blood Mini Kit (Qiagen, Hilden, Germany), according to the manufacturer’s protocol. This method employs a highly efficient silica membrane-based spin column for the rapid purification of DNA from 0.2 mL of blood within a remarkably short time span. The quality and quantity of the resulting DNA samples were evaluated using Nanodrop spectrophotometer (Thermo Fisher Scientific, Waltham) and agarose gel electrophoresis, respectively. Notably, the extracted DNA had an average A260/A280 ratio of 1.8, confirming its purity, with an impressive yield of 6.5 μg per sample. To preserve the integrity of the genetic material, the DNA specimens were preserved at −80°C, pending subsequent analyses of greater significance and depth.

### 2.8. Nested PCR amplification for targeted analysis of specific codons in human DNA sequences

Two amplifications were performed by nested PCR. The first amplification targeted a common outer region of the human genome. For the first run, a Master Mix of 25 μL was prepared from the following: 2.5 mM magnesium chloride (MgCl2; ThermoScientific), 10 mM of each deoxynucleotide triphosphate (dNTP), 2.5 units of DreamTaq DNA polymerase, 10X DreamTaq buffer (Thermoscientific) and nuclease-free water to make 25 μL. 20ng sample DNA was used, and 10 pmol of each forward (5-ACTGACCCCGGTGGCGGAGGA-3) and reverse (5-CAGGCGTATCTGCTGGGCCTGCTC-3) primer (Inqaba Biotec East Africa Ltd., Kampala, Uganda).^[28]^ PCR was performed using the Applied Biosystems SimpliAMp Thermocycler (Thermo Scientific) under the following conditions: 35 cycles of denaturation at 95°C for 30 seconds, annealing at 64°C for 30 seconds, and extension at 72°C for 30 seconds. To ensure complete amplification of all products, a final extension step at 72°C for 7 minutes was performed. The PCR product (5 μL) was run on a 2% agarose gel and viewed under a transilluminator to look for a 514 bp band. The second PCR run targeted codons 112 and 158 of the first amplicon. 5 μL (Could add concentration here, people may be more interested in that than the volume of the internal forward PCR’) and used as-template to for the second PCR using 2.5 μL of 10 pMol of each of the internal forward primers (5′-GGCGCGGACATGGAGGACGgGC-3′) and reverse primers (5′-GCGGTACTGCACCAGGCGGCCtCA-3′) for the 112 codon and the internal forward primers (5′-CGATGCCGATGACCTGCAGAcGC-3′) and reverse primers (5′-CCCGGCCTGGTACACTGCCAGtCA-3′) (Table 1).^[28]^ During the run, 35 cycles of amplification were performed under the following conditions: initial denaturation at 95 ◦C for 5 min, followed by denaturation at 94°C for 30 seconds, annealing at 62°C for 30 seconds, extension at 72°C for 30 seconds, and a final extension at 72°C for 10 minutes. Five microliters of the final PCR product (5 μL) were loaded onto a 2% agarose gel ethidium bromide (1 µg/mL) and electrophoresed at 120 V for 90 minutes. Positive reactions yielded amplicons of 115, 253, 307, and 444 bp (Table 2), which were easily visualized using a UV transilluminator. Nuclease-free cells were used as negative controls.

**Table 1:** Demographic and baseline characteristics.

Variable	AD	Controls	*P* value
n	45	42	
Age (Mean ± SD)	79.62 ± 10.25	73.02 ± 6.64	.0006
Gender, Female (%)	37 (82)	29 (69)	.2363
Education (Median, IQR)	3.0 (0.0–5.0)	4.0 (2.0–7.0)	.1072

**Table 2:** APOE genotype and allele distributions, HWE *P* values, and regression results.

Section	Row_Label	AD	HC	OR_CI	*P* value	Reference
Sample size		n = 45 cases	n = 42 controls	–	–	–
Genotype distribution	ε2/ε3	3 (6.7%)	2 (4.8%)	–	–	–
	ε2/ε4	4 (8.9%)	2 (4.8%)	–	–	–
	ε3/ε3	3 (6.7%)	3 (7.1%)	–	–	Reference
	ε3/ε4	35 (77.8%)	35 (83.3%)	–	–	–
Allele distribution	Allele ε2	7 (7.8%)	4 (4.8%)	–	–	–
	Allele ε3	44 (48.9%)	43 (51.2%)	–	–	–
	Allele ε4	39 (43.3%)	37 (44%)	–	–	–
Hardy-Weinberg equilibrium	HWE *P* value	<.001	<.001	–	–	–
–	ε2/ε3	–	–	0.39 (0.02–5.51)	.49	–
	ε2/ε4	–	–	0.26 (0.02–3.11)	.293	–
	ε3/ε4	–	–	0.58 (0.09–3.64)	.547	–
Regression models		–	–	–	–	–
Logistic regression (APOE*4 dosage)	APOE*4 dosage (0-2 copies)	–	–	0.81 (0.2–3.35)	.763	–
Logistic regression (APOE*2 dosage)	APOE*2 dosage (0-2 copies)	–	–	0.52 (0.11–2.14)	.371	–

*P* values < .05 were considered statistically significant and are highlighted in red. Logistic regression models were adjusted for age, gender, and education. Genotype ε3/ε3 was used as the reference group in all genotype-based regression models. APOE*4 and APOE*2 dosage models represent the additive effect of carrying 0, 1, or 2 alleles.

HWE = Hardy-Weinberg Equilibrium.

### 2.9. Genotyping and quality control

Genotyping of the APOE alleles was performed using PCR-based methods, followed by gel electrophoresis for allele identification. Several quality control (QC) measures have been implemented to ensure data quality. These included duplicate genotyping of a random 10% subset of the samples, which yielded > 99% concordance. Negative controls (no-template reactions) were included in each PCR run to monitor for contamination. In addition, samples with ambiguous or missing genotype calls were excluded from downstream analysis. No batch effects or technical artifacts were observed across the different runs and allele calling was independently verified by two laboratory personnel.

### 2.10. Data analysis

All statistical analyses were conducted using the R software (version 4.3.3) to ensure reproducibility and the use of advanced statistical methods suitable for epidemiological data. Descriptive statistics were used to summarize demographic characteristics, employing means and standard deviations for continuous variables and frequencies and percentages for categorical variables to provide an overview of the population. Independent sample *t* tests, Mann–Whitney *U* tests, and chi-square tests were employed to compare demographic variables between AD and healthy control (HC) groups, allowing for appropriate statistical testing based on variable type and distribution, thus ensuring valid group comparisons.

Logistic regression models were used to assess the association between APOE alleles and AD risk after adjusting for age, education, and sex as covariates. This method was chosen to control for potential confounding factors and quantify the effect of APOE alleles on AD risk. Generalized Additive Models were fitted to explore the nonlinear relationships among age, APOE ε4 status, AD risk, and interaction effects. Generalized Additive Models provide flexibility in modeling complex relationships without assuming linearity, making them ideal for capturing the dynamic effects of age and potential interactions with APOE status and education on AD risk.

Missing data were handled using multiple imputations, which allowed for the estimation of missing values based on observed data patterns, thereby reducing bias and preserving the statistical power. Sensitivity analyses were conducted to compare the results with and without imputed data to ensure the robustness of the findings.

The predicted probabilities of AD were plotted to visualize the relationships, providing an intuitive understanding of how AD risk varies across age groups stratified by APOE allele status and education level. Matching was performed for age and sex to minimize confounding, ensuring that comparisons between the groups were as fair as possible. Analyses were conducted with the significance level set at *P* < .05, providing a conventional threshold for determining statistically significant associations. The results were interpreted by considering model uncertainty, particularly in older age groups with wider confidence intervals, to appropriately account for variability and ensure cautious conclusions.

### 2.11. Genotype distribution and Hardy-Weinberg Equilibrium testing

Hardy-Weinberg Equilibrium (HWE) was assessed separately for AD cases and cognitively normal controls to evaluate whether genotype frequencies conformed to the expected proportions under equilibrium. APOE genotypes were first summarized within each group, and HWE was tested under the assumption of a tri-allelic system (ε2, ε3, and ε4).

The exact test for HWE was performed using the Hardy-Weinberg R package (version 4.3.3), applying a G-test for multi-allelic loci. Deviations from HWE were considered significant at a threshold of *P* < .05. Genotype counts and p-values are reported for each group.

## 3. Results

### 3.1. Demographic and baseline characteristics

A total of 87 participants were included in the study, comprising 45 individuals diagnosed with AD and 42 HC (Table 1). The mean age of the participants in the AD group was 79.62 years (SD = 10.25), which was significantly higher than that of the HC group, with a mean age of 73.02 years (SD = 6.64), with a *P* value of .0006, indicating a statistically significant difference. The proportion of female participants was higher in the AD group (82%) than in the HC group (69%), although the difference was not statistically significant (*P* = .2363). Median years of education were lower in the AD group (3.0 years, IQR: 0.0–5.0) compared to the HC group (4.0 years, IQR: 2.0–7.0), but this difference was not statistically significant (*P* = .1072).

### 3.2. APOE genotype, allele frequencies, and association with AD

The APOE genotype and allele distributions among patients (AD) cases (n = 45) and controls (n = 42) are summarized in Table 2. The most common genotype in both groups was ε3/ε4, which was present in 77.8% of cases and 83.3% of controls. The frequencies of ε2/ε3, ε2/ε4, and ε3/ε3 genotypes were lower and comparable between the groups. The allele frequencies showed that the ε3 allele was the most prevalent, followed by the ε4 and ε2 alleles.

Both the AD and control groups showed significant deviations from Hardy-Weinberg Equilibrium (*P* < .001). No statistically significant associations were observed between APOE genotypes and AD status in the logistic regression models adjusted for age, sex, and education. Compared to the ε3/ε3 reference group, none of the genotypes (ε2/ε3, ε2/ε4, or ε3/ε4) was associated with altered odds of AD (all *P* > .05). Similarly, the APOE*4 and APOE*2 allele dosage models (reflecting 0–2 copies) did not show significant associations with AD risk.

### 3.3. Comparison of APOE allele frequencies with 1000 genomes African ancestry data

Compared to the 1000 Genomes Project African ancestry data, the APOE allele frequencies in both the AD (CA) and control (CO) groups demonstrated elevated ε4 frequencies. The ε4 allele was observed in 42.2% of the AD group and 44.0% of the control group, which is considerably higher than the reported ε4 frequencies in African ancestry populations from the 1000 Genomes Project (typically 10–15%). Similarly, the ε3 allele frequency was lower in both groups (50.0% in AD and 51.2% in controls) than in the reference (~80%). The ε2 allele was detected at 7.8% in the AD group and 4.8% in the control group, slightly below the reference frequency (~9%).

### 3.4. Interaction between age and APOE ε4 carrier status and AD risk

**Figure 1.**
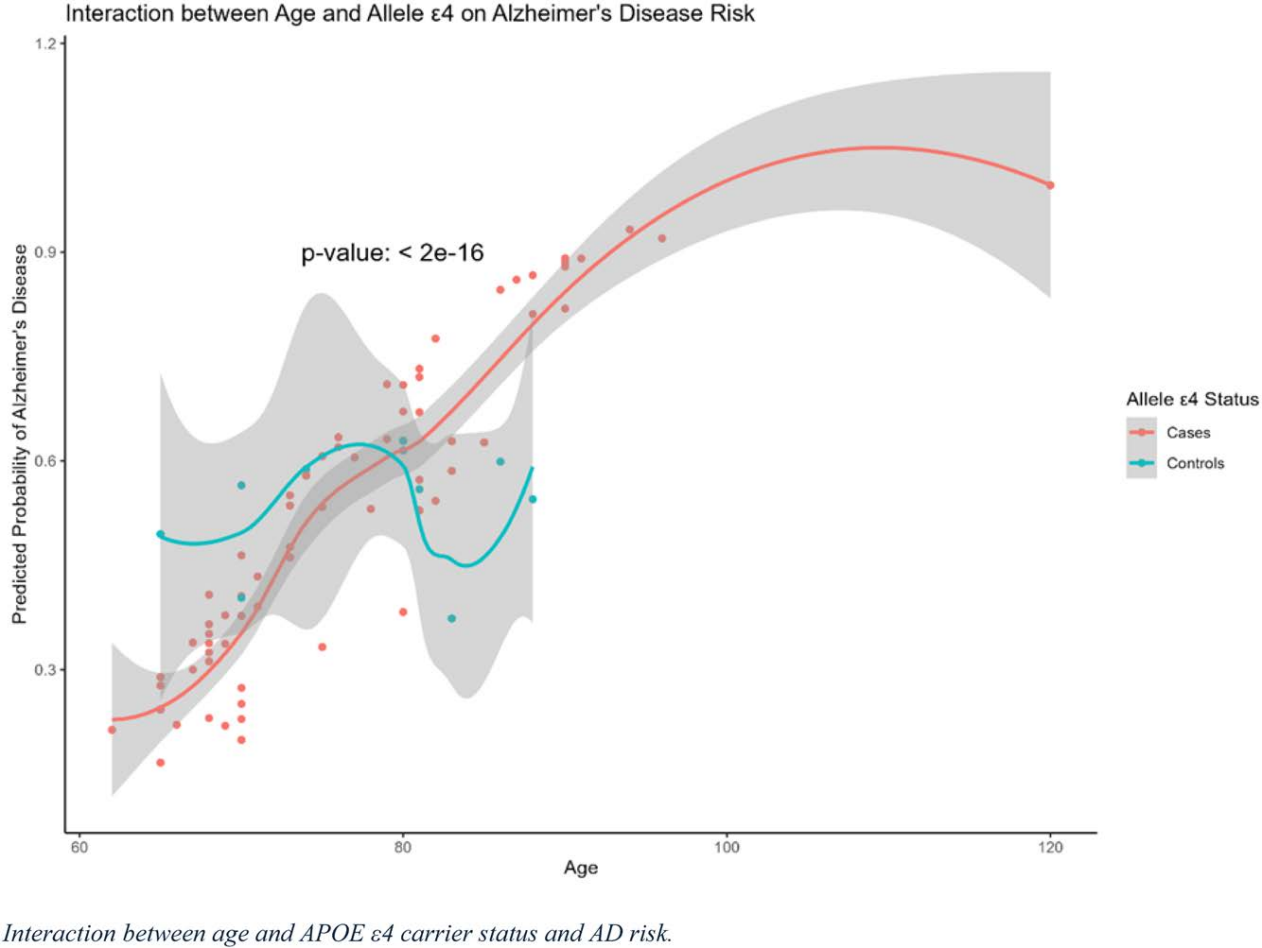
The interaction effect between age and APOE ε4 on the predicted probability of developing Alzheimer’s disease (AD) risk and the interaction effect between age and APOE ε4 carrier status on the predicted probability of developing AD. The x-axis represents age, and the y-axis indicates the predicted probability of AD. The solid line represents ε4 carriers (cases), whereas the dashed line represents non-carriers (controls). The shaded areas around the lines indicate 95% confidence intervals.

The interaction between age and APOE ε4 allele status on the predicted probability of Alzheimer’s is shown in Figure 1. The red line represents individuals carrying the ε4 allele among the cases, whereas the blue line represents controls without the ε4 allele. The predicted probability of Alzheimer’s increases with age, with a notably higher probability in ε4 carriers. The curve for ε4 carriers showed a consistent upward trend, reaching a probability close to 1 in older individuals, indicating a strong association between advancing age and increased risk of AD in this group. In contrast, the control group showed more variation, with a relatively lower predicted probability of AD across the age spectrum. The shaded regions represent confidence intervals, and the *P* value for the interaction was highly significant (*P* < .05), suggesting a significant effect of the interaction among age, APOE ε4 carrier status, and AD risk.

### 3.5. Predicted probability of AD based on age and APOE ε2 allele status

**Figure 2.**
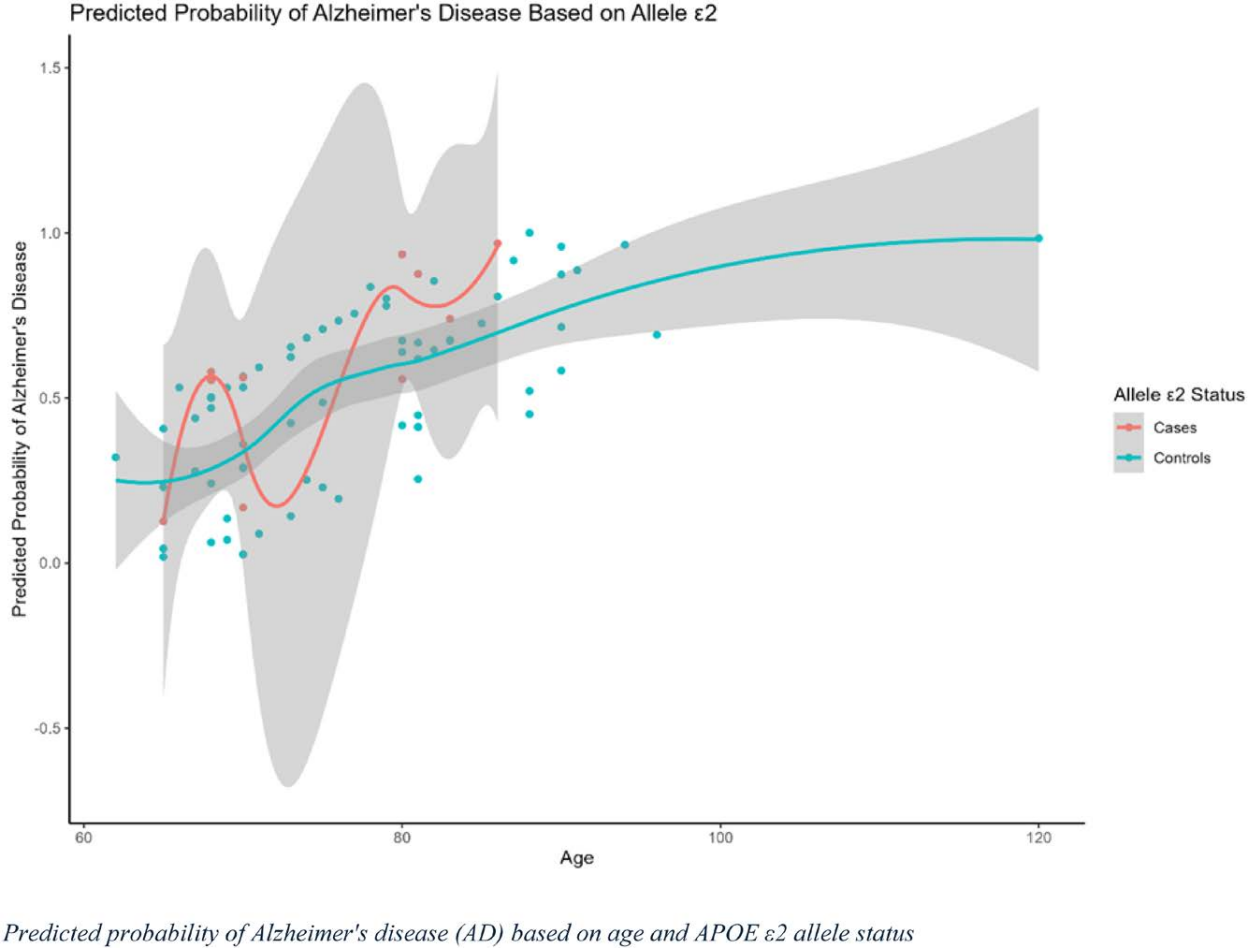
Predicted probability of Alzheimer’s disease (AD) based on APOE ε2 carrier status. This graph shows the predicted probability of developing AD in relation to age and APOE ε2 carrier status. The x-axis represents age, whereas the y-axis represents the predicted probability of AD. The solid curve represents individuals carrying the APOE ε2 allele (cases), whereas the dashed curve represents non-carriers (controls). The shaded areas around the curves indicate 95% confidence intervals for the predicted probabilities.

The predicted probability of AD based on age and APOE ε2 allele status is depicted in Figure 2. The red line represents individuals with AD carrying the ε2 allele, whereas the blue line represents healthy controls carrying the ε2 allele. The predicted probability of AD showed notable fluctuations across age among ε2 carriers in the AD group with no clear upward trend. Conversely, the control group demonstrated a more stable pattern, with a relatively lower probability of AD across the age spectrum. Overall, the presence of the ε2 allele appeared to be associated with a more variable and potentially protective effect on AD risk than the other alleles, as indicated by the lower predicted probability in the control group. The shaded regions indicate confidence intervals, reflecting the degree of uncertainty in the model’s predictions, which is particularly pronounced for the AD group across certain age ranges.

### 3.6. Predicted probability of AD in relation to age and APOE ε3 allele status

**Figure 3.**
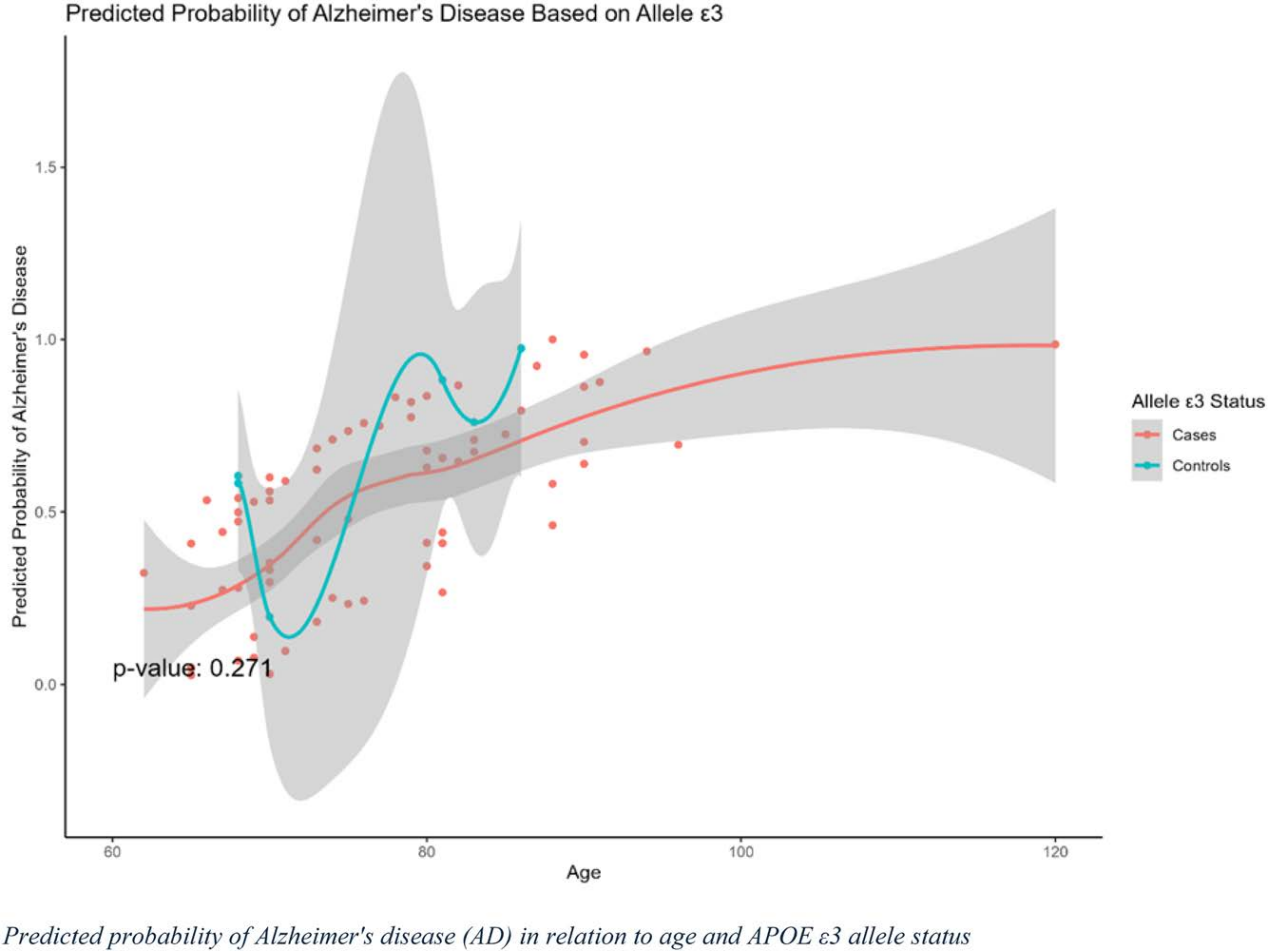
Predicted probability of Alzheimer’s disease (AD) based on APOE ε3 carrier status. This graph depicts the predicted probability of developing AD in relation to age and APOE ε3 carrier status. The x-axis represents age, whereas the y-axis represents the predicted probability of AD. The solid curve represents individuals carrying the APOE ε3 allele (cases), whereas the dashed curve represents non-carriers (controls). The shaded areas around the curves indicate 95% confidence intervals for the predicted probability estimates.

The predicted probability of AD in relation to age and APOE ε3 allele status is shown in Figure 3. The results indicated that the predicted probability of AD among ε3 carriers did not show a significant difference between cases and controls, as indicated by a *P* value of .271. For both groups, the probability trends remained stable across the age range with some fluctuations, particularly in the control group. The wide shaded region representing the confidence intervals suggests variability and a lack of definitive relationship between ε3 carrier status and AD risk. Overall, these findings suggest that the presence of the ε3 allele is not significantly associated with an increased risk of AD and no clear age-dependent effect was observed for this allele.

### 3.7. Predicted probability of AD as a function of age, APOE ε4 carrier status, and education level

**Figure 4.**
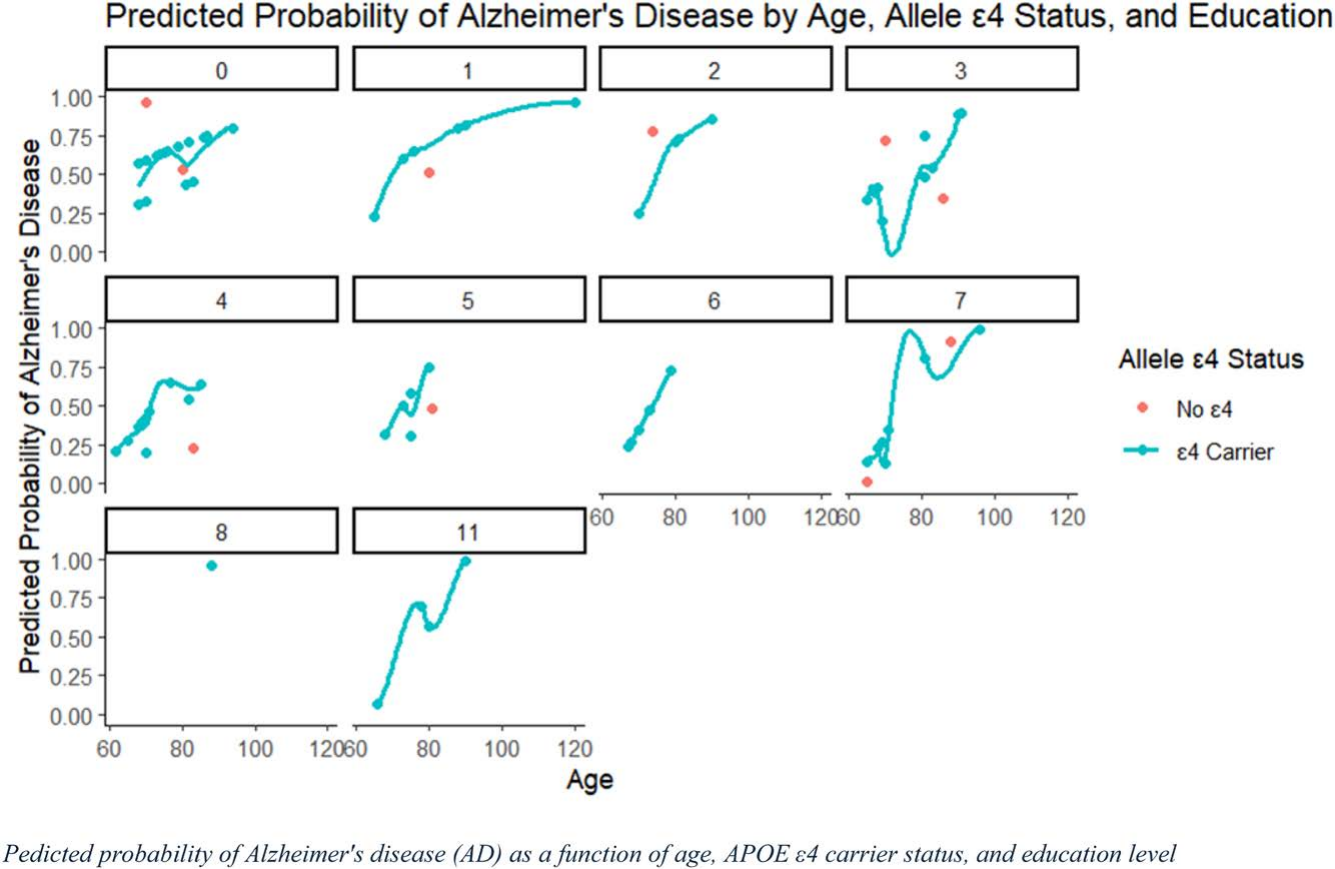
Predicted probability of Alzheimer’s disease (AD) by age, APOE ε4 status, and education level: This illustrating the predicted probability of developing AD based on age, APOE ε4 status, and education level. The x-axis represents age, and the y-axis indicates the predicted probability of AD. Each panel represents a different educational level (0, 1, 2, 3, 4, 5, 6, 7, 8, and 11 years), highlighting the effect of education on AD risk.

The predicted probabilities of AD as a function of age, APOE ε4 carrier status, and education level are shown in Figure 4. The figure includes individual panels representing different levels of education ranging from 0 to 11 years. Within each panel, the blue line represents individuals who are ε4 carriers, while red dots indicate individuals without the ε4 allele. The predicted probability of AD generally increased with age across most educational levels, particularly among ε4 carriers. This figure suggests a positive interaction between age and ε4 carrier status in predicting AD risk, with higher predicted probabilities of AD observed among ε4 carriers, particularly at older ages and lower educational levels.

The effect of education appears inconsistent across panels, with fluctuations depending on the age and ε4 status. For individuals with higher education (e.g., 8 or more years), the predicted probability of AD remains lower than that for those with minimal education, suggesting a possible protective effect of education against AD. These findings indicate that APOE ε4 carrier status, age, and education level interact to influence the probability of AD, with ε4 carriers and individuals with lower education levels being at a higher risk.

### 3.8. Predicted probability of AD as a function of age

**Figure 5.**
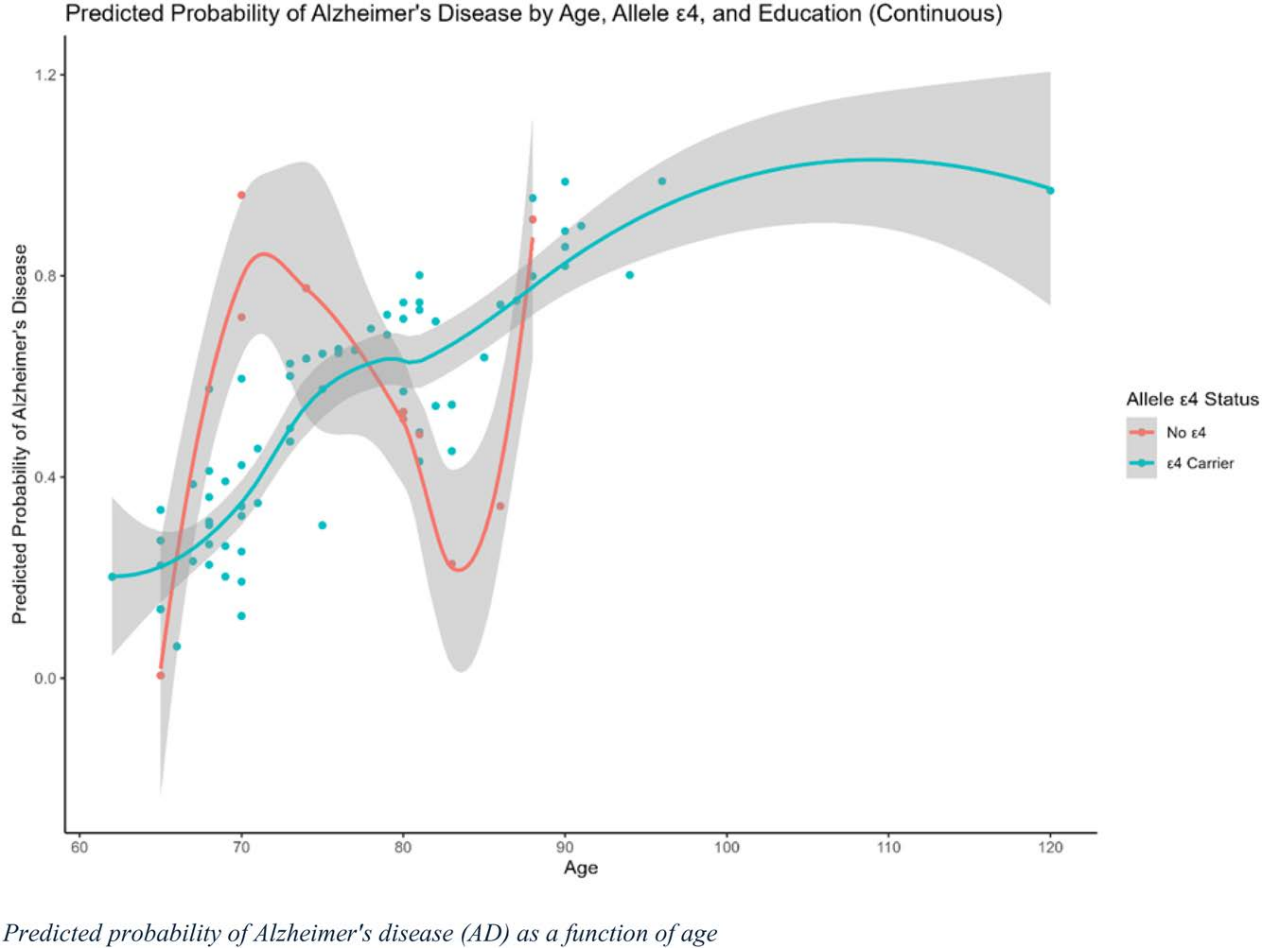
Predicted probability of Alzheimer’s disease (AD) by Age, APOE ε4 Status, and education level (continuous): This graph illustrates the relationship between age, APOE ε4 status, and the predicted probability of developing AD, with education level treated as a continuous variable. The x-axis represents age, whereas the y-axis represents the predicted probability of AD. The solid curve represents individuals without the APOE ε4 allele (non-carriers), whereas the dashed curve represents ε4 carriers. The shaded regions around each curve indicate 95% confidence intervals for the predicted probabilities.

The predicted probability of AD as a function of age, APOE ε4 carrier status, and education level (treated as a continuous variable) is shown in Figure 5. The red line represents individuals who did not carry the ε4 allele, whereas the blue line represents ε4 carriers. The shaded regions denote the confidence intervals for the predictions. The figure shows that the predicted probability of AD increased with age in both ε4 carriers and non-carriers, although the patterns differed between the two groups. For ε4 carriers, the predicted probability gradually increases with age, particularly after the age of 80. In contrast, the predicted probability for non-carriers exhibits more pronounced fluctuations, with a notable increase between ages 70 and 80, followed by a leveling off.

The confidence intervals were wider for older age groups, indicating greater uncertainty in the predictions for these age ranges. The influence of ε4 carrier status on AD risk appears to be less prominent at younger ages but becomes more pronounced with advancing age, as evidenced by the divergence of the curves. These results suggest that age is a significant risk factor for AD, with ε4 carriers demonstrating a higher probability of developing AD with increasing age. The continuous representation of education did not show a clear protective effect in this interaction but highlighted variability in the risk of AD among different age groups and APOE ε4 status.

### 3.9. Predicted probability of AD as a function of age, adjusted for education and sex, and stratified by APOE ε4 allele status

**Figure 6.**
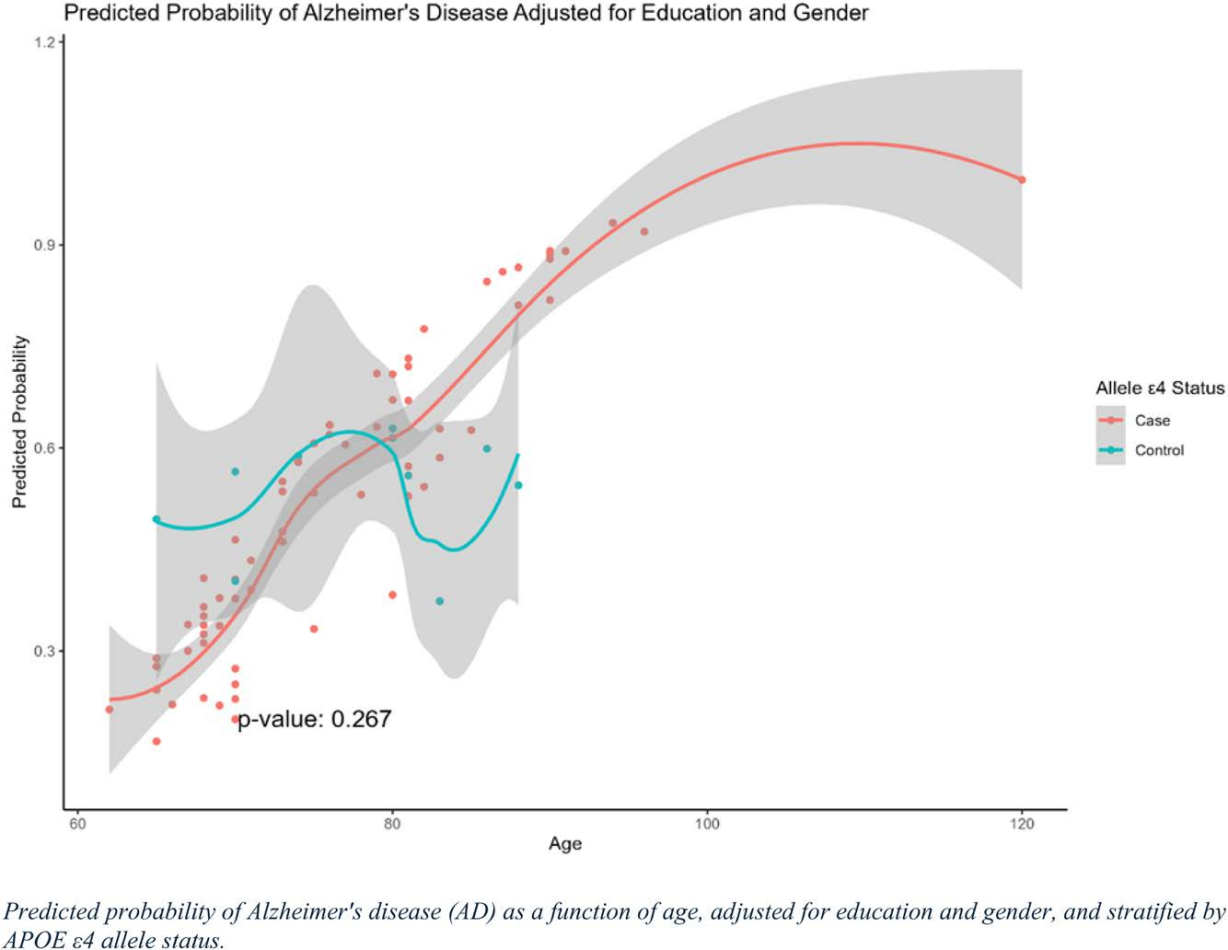
Predicted probability of Alzheimer’s disease (AD) adjusted for education and sex. This graph represents the predicted probability of AD in relation to age and APOE ε4 carrier status, adjusted for education and sex. The x-axis indicates age, and the y-axis shows the predicted probability of AD. The solid curve represents individuals carrying the APOE ε4 allele (AD), whereas the dashed curve represents non-carriers (controls). The shaded regions around the curves represent 95% confidence intervals for the predicted probabilities.

The predicted probability of AD as a function of age, adjusted for education and sex, and stratified by APOE ε4 allele status is shown in Figure 6. The red line represents individuals with AD (cases), whereas the blue line represents the healthy controls. The figure shows that the predicted probability of AD increased with age in both groups, with a more pronounced increase observed in the AD group. The predicted probability of AD among patients starts to increase more rapidly around the age of 75 years and continues to increase steadily with age, reaching approximately 1.0. In contrast, controls exhibited a less consistent pattern, with a moderate increase in AD probability and some fluctuations, particularly between 70 and 90 years of age.

A *P* value of .267 suggested that the interaction between age and ε4 carrier status, adjusted for education and sex, was not statistically significant. This indicates that, while age remains a strong predictor of AD risk, the additional effect of ε4 status is less evident in this adjusted model. The shaded regions representing the confidence intervals were wider at older ages, reflecting greater variability and uncertainty in the predicted probabilities, particularly in the AD group.

### 3.10. Predicted probability of AD as a function of age adjusted for education and sex

**Figure 7.**
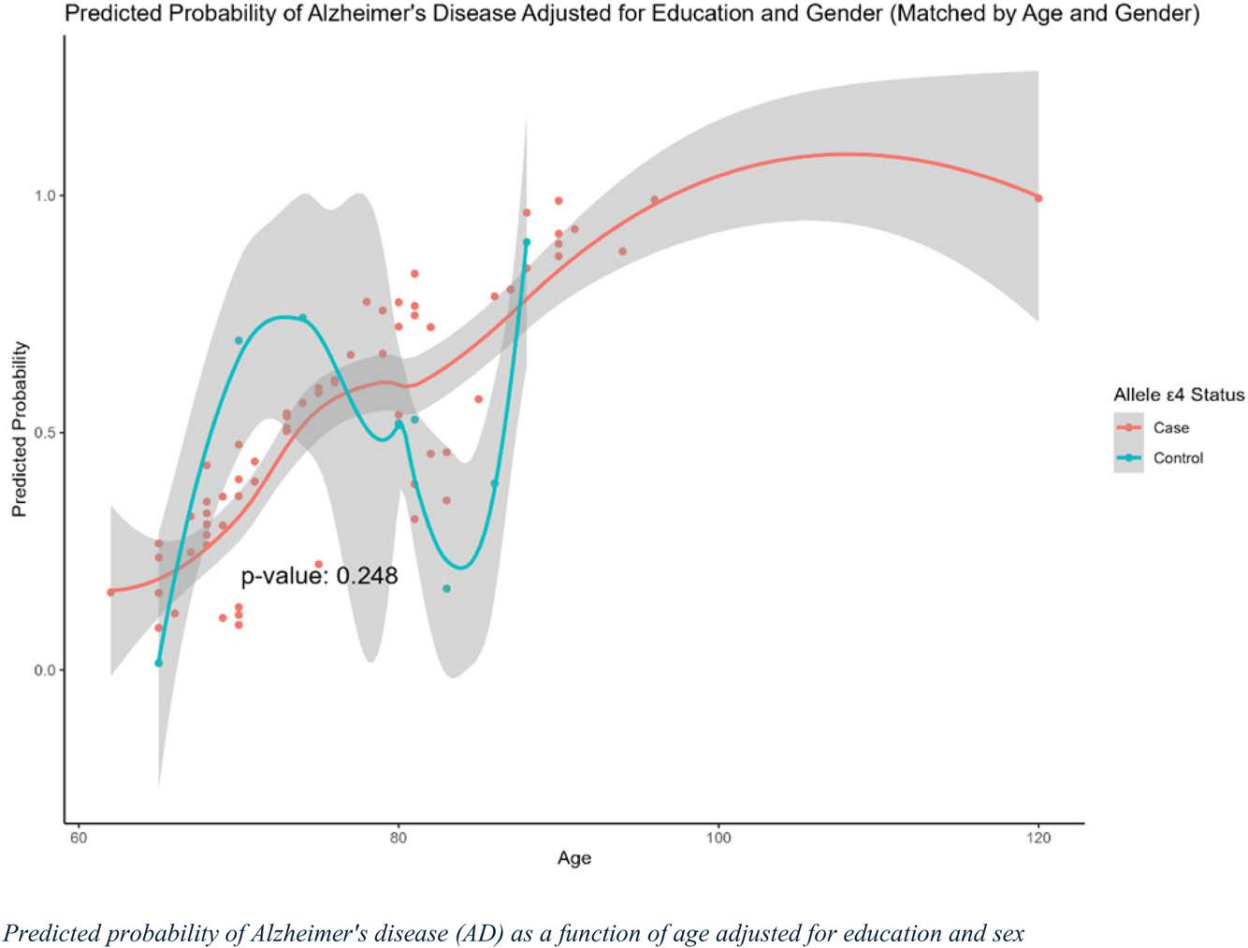
Predicted probability of Alzheimer’s disease (AD) by age, adjusted for education and sex (matched by age and sex). This graph shows the predicted probability of AD as a function of age, adjusted for education and sex, with matching by age and sex to minimize confounding effects. The x-axis represents age, and the y-axis indicates the predicted probability of AD. The solid line represents individuals carrying the APOE ε4 allele (AD), whereas the dashed line represents non-carriers (controls). The shaded regions around each line represent 95% confidence intervals for the predicted probabilities.

The predicted probability of AD as a function of age, adjusted for education and sex, with the analysis matched by age and sex, is shown in Figure 7. The red line represents individuals with AD (cases), whereas the blue line represents the healthy controls. The shaded regions indicate the confidence intervals for each group. The figure demonstrates an increasing predicted probability of AD with age for both groups, with AD cases generally showing a higher predicted probability than the controls. The control group exhibited greater fluctuations in predicted probability, particularly between the ages of 70 and 90 years, whereas the AD group showed a relatively steady increase, particularly after the age of 80 years. The divergence between the two groups became more apparent as age advanced.

A *P* value of .248 indicated no statistically significant difference between the case and control groups in the interaction between age and APOE ε4 carrier status, after adjusting for education and sex. The wide confidence intervals at older ages reflected the variability and uncertainty in the model’s predictions, particularly for the control group. Overall, this trend suggests that APOE ε4 carrier status, combined with older age, may be associated with an elevated risk of AD, although this difference was not statistically significant in the adjusted and matched models.

## 4. Discussion

The findings of this study provide further insights into the relationship between apolipoprotein E (APOE) allele status and AD risk in the Ugandan population, highlighting both common and unique patterns compared to other regions. Notably, the APOE ε4 allele, commonly associated with an elevated risk of AD, was observed at a similar frequency in both groups, accounting for 43.3% of the alleles among AD cases and 44% among controls. This lack of a statistically significant difference aligns with previous research in sub-Saharan African populations, where the association between ε4 and AD risk varied. These findings suggest that the effect of APOE ε4 status on AD risk may be modulated by additional factors in African populations, including genetic background, lifestyle, and environmental exposure.^[20,29]^ These results underscore the importance of population-specific investigations in interpreting the role of APOE in AD pathogenesis.

Interestingly, we observed that the ε2 allele, generally regarded as protective against AD, was slightly more frequent among AD cases (7.8%) than in healthy controls (4.8%). While this observation diverges from the typical pattern reported in many non-African populations, where ε2 is often underrepresented among AD cases, it highlights the complex and potentially population-specific role of APOE ε2 in AD pathogenesis.^[9,30]^ Despite its modest frequency difference, the presence of ε2 did not show a statistically significant protective effect in our logistic regression analysis, possibly due to the limited sample size or the influence of other confounding factors, such as genetic admixture, lifestyle differences, or comorbid health conditions. Mechanistically, APOE ε2 has been proposed to facilitate Aβ clearance and reduce neuroinflammation; however, such protective effects may vary across populations. Although age emerged as a significant predictor of AD risk in our study, the interaction between age and APOE ε4 carrier status was not significant after adjusting for sex and education. This suggests that the influence of APOE ε4 status on AD risk may be less pronounced in this Ugandan cohort than in other populations. Larger studies are needed to confirm these findings and explore the interplay between APOE variants and other population-specific factors.

The predicted probability of AD increases with age in both ε4 carriers and non-carriers, with ε4 carriers generally exhibiting a higher probability of AD. This trend was particularly evident among individuals with lower education levels, suggesting that education may play a role in modulating AD risk, potentially through cognitive reserve mechanisms.^[31,32]^ Cognitive reserve refers to the resilience of the brain against neuropathological damage. Higher education levels may enhance cognitive reserve, allowing individuals to better compensate for the effects of AD-related changes.^[31]^ Therefore, APOE ε4 carriers with higher cognitive reserves may experience delayed onset or reduced symptom severity compared with those with lower cognitive reserves. However, the effect of education on AD risk appeared inconsistent, with fluctuations observed across different age groups and APOE statuses. This inconsistency could be attributed to factors such as varying quality of education, socio-economic disparities, and differences in access to healthcare.^[33,34]^ Future studies should address these variables using more detailed measures of educational quality and socioeconomic status to better understand their influence on AD risk. The variability in the impact of education and lack of significant protective effects among those with higher education levels warrant further investigation to better understand the socio-environmental factors at play.

Our study emphasizes the importance of expanding genetic research to underrepresented populations, as the variability in APOE allele impact across African populations highlights the complex interplay between genetics and environment in AD pathogenesis.^[35,36]^ Specific environmental factors, such as diet, infectious disease burden, physical activity, and socioeconomic conditions, may interact with genetic predispositions to influence AD risk in these populations.^[37]^ While most research on APOE and AD has focused on European or Asian populations, our findings provide valuable insights into the genetic epidemiology of AD in sub-Saharan Africa. These insights are crucial for developing targeted interventions that are culturally and genetically appropriate for the African population. Further studies are required to elucidate the role of additional genetic variants, such as ABCA7, SORL1, and TREM2, and their environmental influences, including diet, physical activity, and chronic stress, which may contribute to the risk of AD in this population.

Overall, this study highlights the critical need for future studies with larger sample sizes to better understand the relationship between the APOE ε4 status and AD. The lack of strong association observed in this cohort may be due to the limited sample size. A larger cohort would enable more robust analyses, allowing for the examination of interactions between genetic factors, such as APOE ε4, and other modulating factors, such as education and age. Future studies with larger sample sizes are essential to validate these findings and to provide a clearer understanding of the genetic and environmental contributions to AD risk.

### 4.1. Strengths of the study

This study investigated APOE allele frequencies and their impact on AD in the understudied Ugandan population using advanced statistical methods to adjust for confounders. These findings suggest that cognitive reserve, influenced by education, may modulate AD risk, offering valuable insights into genetic and socio-environmental factors in sub-Saharan Africa.

### 4.2. Limitations

The relatively small sample size limits the statistical power and restricts the generalizability of our findings to the broader Ugandan and sub-Saharan African population. Additionally, the case-control design precludes causal inferences regarding the relationship between APOE alleles and AD risk. Although we excluded closely related individuals based on their family history, formal genotypic assessments for cryptic relatedness were not performed, leaving the possibility of distant relatedness within the sample. The use of self-reported data introduces potential recall bias, and residual confounding from unmeasured factors such as socioeconomic status, comorbidities, or lifestyle may have influenced the results.

Another limitation is the deviation from the Hardy-Weinberg Equilibrium observed in both the case and control groups. While deviation in the AD group could reflect a true genetic association, deviation in the control group suggests the presence of a population substructure, sample stratification, or technical artifacts. This may limit the interpretability and generalizability of the genetic findings. Future studies should recruit larger and more diverse cohorts, apply longitudinal designs, and incorporate genotyping to adjust for population stratification and relatedness with validation in independent datasets.

### 4.3. Generalizability of the study results

The generalizability of this study is limited by the specific characteristics of the Ugandan population and the rural-urban setting. Although the diverse demographic composition improves applicability to similar sub-Saharan African populations, the small sample size (87 participants) and selection of controls from participants’ spouses may limit the broader relevance. Comparable frequencies of the APOE ε4 allele between AD cases and controls suggest that the impact of APOE ε4 may differ in this population, possibly due to unique genetic and environmental factors. The inconsistent effects of education on AD risk also limit generalizability. Larger and more diverse studies are needed to confirm these findings.

## Acknowledgments

We express our sincere gratitude to Ms. Sylvia Nalwanga for her invaluable support and assistance throughout this study.

## Author contributions

**Conceptualization:** Kamada Lwere, Haruna Muwonge, Joy Louise Gumukiriza-Onoria, William Buwembo, Ian Guyton Munabi.

**Data curation:** Kamada Lwere, Joy Louise Gumukiriza-Onoria, William Buwembo, Aisha Nazziwa, Ian Guyton Munabi.

**Formal analysis:** Kamada Lwere, Haruna Muwonge, Joy Louise Gumukiriza-Onoria, Denis Buwembo, William Buwembo, Rita Nassanga, Aisha Nazziwa, Ian Guyton Munabi.

**Funding acquisition:** Kamada Lwere, William Buwembo, Mark Kaddumukasa.

**Investigation:** Kamada Lwere, Haruna Muwonge.

**Methodology:** Kamada Lwere, Haruna Muwonge, Joy Louise Gumukiriza-Onoria, William Buwembo.

**Project administration:** Kamada Lwere.

**Software:** Kamada Lwere.

**Supervision:** Haruna Muwonge, Hakim Sendagire, Noeline Nakasujja, Mark Kaddumukasa.

**Validation:** Kamada Lwere.

**Visualization:** Kamada Lwere.

**Writing – original draft:** Kamada Lwere, Haruna Muwonge, Joy Louise Gumukiriza-Onoria, Denis Buwembo, Rita Nassanga, Rheem Nakimbugwe, Aisha Nazziwa, Ian Guyton Munabi.

**Writing – review & editing:** Kamada Lwere, Haruna Muwonge, Martha Sajatovic, Joy Louise Gumukiriza-Onoria, William Buwembo, Ian Guyton Munabi, Noeline Nakasujja, Mark Kaddumukasa.

## References

[1] World Health Organization. Dementia. https://www.who.int/news-room/fact-sheets/detail/dementia. Accessed October, 2024.

[2] Scarmeas N. Physical activity, diet, and risk of Alzheimer disease. JAMA. 2009;302:627–37. 19671904 10.1001/jama.2009.1144 PMC2765045

[3] Yamazaki Y Zhao N Caulfield TR Liu CC Bu G. Apolipoprotein E and Alzheimer disease: pathobiology and targeting strategies. Nat Rev Neurol. 2019;15:501–18. 31367008 10.1038/s41582-019-0228-7 PMC7055192

[4] Bu G. Apolipoprotein E and its receptors in Alzheimer’s disease: pathways, pathogenesis and therapy. Nat Rev Neurosci. 2009;10:333–44. 19339974 10.1038/nrn2620 PMC2908393

[5] Husain MA Laurent B Plourde M. APOE and Alzheimer’s disease: from lipid transport to physiopathology and therapeutics. Front Neurosci. 2021;15:630502. 33679311 10.3389/fnins.2021.630502 PMC7925634

[6] Shin KC Ali Moussa HY Park Y. Cholesterol imbalance and neurotransmission defects in neurodegeneration. Exp Mol Med. 2024;56:1685–90. 39085348 10.1038/s12276-024-01273-4 PMC11371908

[7] Sun Y-Y Wang Z Huang H-C. Roles of apoE4 on the pathogenesis in Alzheimer’s disease and the potential therapeutic approaches. Cell Mol Neurobiol. 2023;43:3115–36. 37227619 10.1007/s10571-023-01365-1 PMC10211310

[8] Nelson PT Pious NM Jicha GA. APOE-ε2 and APOE-ε4 correlate with increased amyloid accumulation in cerebral vasculature. J Neuropathol Exp Neurol. 2013;72:708–15. 23771217 10.1097/NEN.0b013e31829a25b9 PMC3715146

[9] Li Z Shue F Zhao N Shinohara M Bu G. APOE2: protective mechanism and therapeutic implications for Alzheimer’s disease. Mol Neurodegener. 2020;15:63. 33148290 10.1186/s13024-020-00413-4 PMC7640652

[10] de-Almada BV de-Almeida LD Camporez D. Protective effect of the APOE-e3 allele in Alzheimer’s disease. Braz J Med Biol Res. 2012;45:8–12. 22068907 10.1590/S0100-879X2011007500151 PMC3854133

[11] Kim J Basak JM Holtzman DM. The role of apolipoprotein E in Alzheimer’s disease. Neuron. 2009;63:287–303. 19679070 10.1016/j.neuron.2009.06.026 PMC3044446

[12] Shi Y Yamada K Liddelow SA; Alzheimer’s Disease Neuroimaging Initiative. ApoE4 markedly exacerbates tau-mediated neurodegeneration in a mouse model of tauopathy. Nature. 2017;549:523–7. 28959956 10.1038/nature24016 PMC5641217

[13] Safieh M Korczyn AD Michaelson DM. ApoE4: an emerging therapeutic target for Alzheimer’s disease. BMC Med. 2019;17:64. 30890171 10.1186/s12916-019-1299-4 PMC6425600

[14] Raulin AC Doss SV Trottier ZA Ikezu TC Bu G Liu CC. ApoE in Alzheimer’s disease: pathophysiology and therapeutic strategies. Mol Neurodegener. 2022;17:72. 36348357 10.1186/s13024-022-00574-4 PMC9644639

[15] Hall K Murrell J Ogunniyi A. Cholesterol, APOE genotype, and Alzheimer disease: an epidemiologic study of Nigerian Yoruba. Neurology. 2006;66:223–7. 16434658 10.1212/01.wnl.0000194507.39504.17 PMC2860622

[16] Boyce MR Katz R Standley CJ. Risk factors for infectious diseases in urban environments of sub-Saharan Africa: a systematic review and critical appraisal of evidence. Trop Med Infect Dis. 2019;4:123. 31569517 10.3390/tropicalmed4040123 PMC6958454

[17] Le Guen Y Raulin A-C Logue MW. Association of African ancestry–specific APOE missense variant R145C with risk of Alzheimer disease. JAMA. 2023;329:551–60. 36809323 10.1001/jama.2023.0268 PMC9945061

[18] Ray NR Kunkle BW Hamilton‐Nelson KL. Extended genome‐wide association study employing the African genome resources panel identifies novel susceptibility loci for Alzheimer’s disease in individuals of African ancestry. Alzheimers Dement. 2024;19:5247–61. 10.1002/alz.13880 PMC11350055 38958117

[19] Belloy ME Andrews SJ Le Guen Y. APOE genotype and Alzheimer disease risk across age, sex, and population ancestry. JAMA Neurol. 2023;80:1284–94. 37930705 10.1001/jamaneurol.2023.3599 PMC10628838

[20] Hendrie HC Murrell J Baiyewu O. APOE ε4 and the risk for Alzheimer disease and cognitive decline in African Americans and Yoruba. Int Psychogeriatr. 2014;26:977–85. 24565289 10.1017/S1041610214000167 PMC4012422

[21] Rajabli F Beecham GW Hendrie HC.; Alzheimer’s Disease Sequencing Project, Alzheimer’s Disease Genetic Consortium. A locus at 19q13. 31 significantly reduces the ApoE ε4 risk for Alzheimer’s Disease in African Ancestry. PLoS Genet. 2022;18:e1009977. 35788729 10.1371/journal.pgen.1009977 PMC9286282

[22] Uganda Bureau of Statistics (UBOS). National Population and Housing Census 2014: Main Report. Uganda Bureau of Statistics; 2016. https://www.ubos.org/wp-content/uploads/publications/03_20182014_National_Census_Main_Report.pdf. Accessed October 2024.

[23] Gumikiriza-Onoria J Nakigudde J Mukasa MK Tibasiima I Mayega R Nakasujja N. Reflections of ageing among older adults in a Ugandan community: a qualitative analysis into the benefits and pains of ageing. J Ment Health Aging (Lond). 2023;7:171. 38288055 PMC10824533

[24] Ilardi CR Menichelli A Michelutti M Cattaruzza T Manganotti P. Optimal MoCA cutoffs for detecting biologically-defined patients with MCI and early dementia. Neurol Sci. 2023;44:159–70. 36169756 10.1007/s10072-022-06422-z PMC9816212

[25] Nasreddine ZS Phillips NA Bédirian V. The montreal cognitive assessment, MoCA: a brief screening tool for mild cognitive impairment. J Am Geriatr Soc. 2005;53:695–9. 15817019 10.1111/j.1532-5415.2005.53221.x

[26] W. H. Organization. International Classification of Diseases for Mortality and Morbidity Statistics. 11th ed. https://icd.who.int/browse/2024-01/mms/en. Accessed October, 2024.

[27] American Psychiatric Association. Diagnostic and Statistical Manual of Mental Disorders. 5th ed. American Psychiatric Publishing; 2013. https://psycnet.apa.org/record/2013-14907-000. Accessed November 2024.

[28] Yang YG Kim JY Park SJ Kim SW Jeon OH Kim DS. Apolipoprotein E genotyping by multiplex tetra-primer amplification refractory mutation system PCR in single reaction tube. J Biotechnol. 2007;131:106–10. 17643539 10.1016/j.jbiotec.2007.06.001

[29] Rajabli F Feliciano BE Celis K. Ancestral origin of ApoE ε4 Alzheimer disease risk in Puerto Rican and African American populations. PLoS Genet. 2018;14:e1007791. 30517106 10.1371/journal.pgen.1007791 PMC6281216

[30] Corder EH Saunders AM Strittmatter WJ. Gene dose of apolipoprotein E type 4 allele and the risk of Alzheimer’s disease in late onset families. Science. 1993;261:921–3. 8346443 10.1126/science.8346443

[31] Stern Y. Cognitive reserve in ageing and Alzheimer’s disease. Lancet Neurol. 2012;11:1006–12. 23079557 10.1016/S1474-4422(12)70191-6 PMC3507991

[32] Meng X D’Arcy C. Education and dementia in the context of the cognitive reserve hypothesis: a systematic review with meta-analyses and qualitative analyses. PLoS One. 2012;7:e38268. 22675535 10.1371/journal.pone.0038268 PMC3366926

[33] Langa KM Larson EB Crimmins EM. A comparison of the prevalence of dementia in the United States in 2000 and 2012. JAMA Intern Med. 2017;177:51–8. 27893041 10.1001/jamainternmed.2016.6807 PMC5195883

[34] Valenzuela MJ Sachdev P. Brain reserve and dementia: a systematic review. Psychol Med. 2006;36:441–54. 16207391 10.1017/S0033291705006264

[35] Rotimi CN Tekola-Ayele F Baker JL Shriner D. The African diaspora: history, adaptation and health. Curr Opin Genet Dev. 2016;41:77–84. 27644073 10.1016/j.gde.2016.08.005 PMC5318189

[36] Guerchet M Mayston R Lloyd-Sherlock P. Dementia in sub-Saharan Africa: Challenges and opportunities. Alzheimer’s Disease International; 2017.

[37] Kalaria RN Maestre GE Arizaga R.; World Federation of Neurology Dementia Research Group. Alzheimer’s disease and vascular dementia in developing countries: prevalence, management, and risk factors. Lancet Neurol. 2008;7:812–26. 18667359 10.1016/S1474-4422(08)70169-8 PMC2860610

